# Duffy‐null erythrocyte phenotype and the risk of thrombotic events in essential thrombocythemia

**DOI:** 10.1002/jha2.773

**Published:** 2023-08-17

**Authors:** Ivan Krecak, Marko Lucijanic

**Affiliations:** ^1^ Department of Internal Medicine General Hospital of Sibenik‐Knin County Sibenik Croatia; ^2^ Faculty of Medicine University of Rijeka Rijeka Croatia; ^3^ Division of Hematology University Hospital Dubrava Zagreb Croatia; ^4^ Faculty of Medicine University of Zagreb Zagreb Croatia

Dear Editor‐in‐Chief,

With great interest, we have read the recent article by Karrar et al. [1], published in the *British Journal of Haematology*, which investigated the impact of blood group type on clinical outcomes in patients with essential thrombocythemia (ET). The authors have reported that patients with B‐type blood group were more prone to venous thrombosis than patients with other blood groups; this effect was independent of antiplatelet and cytoreductive treatment and was confined to males. We would like to point out that even though ET patients with B‐type blood group more frequently received cytoreductive and anticoagulant treatment in comparison to patients with other blood groups (Table 1 in the original article), they suffered from more venous events [1]. This interesting observation deserves a comment and may suggest that other underlying pathophysiological mechanisms could be responsible for the reported phenomenon.

The Duffy blood group system, also known as Duffy antigen receptor for chemokines (DARC), is a highly immunogenic glycoprotein antigen complex located on the surface of erythrocytes, vascular endothelial cells, alveolar epithelial cells, kidney tubular cells, and the Purkinje cells in the brain [2]. Globally, the Duffy‐negative phenotype predominates across sub‐Saharan Africa [3] and is of particular importance due to its intrinsic resistance to *Plasmodium vivax* infection [4]. DARC also serves as a chemokine receptor decoy; it binds and internalizes different chemokines responsible for leukocyte trafficking and tissue infiltration [2, 5]. In fact, DARC‐negative status has been found to be responsible for benign ethnic neutropenia, a chronic congenital form of mild neutropenia (without the tendency for infections) in persons of African, Middle Eastern, and West Indian ancestry [6]. Pathophysiologically, enhanced leukocyte trafficking secondary to DARC absence and high chemokine signaling in the circulation leads to increased tissue sequestration of leukocytes, mostly in the spleen [2, 5]. Finally, through its role in maintaining the inflammatory homeostasis in the organism, DARC may also dampen inflammation‐linked carcinogenesis and prevent development and progression of different cancers [5, 7].

We congratulate the authors on providing more insight into the pathogenesis of thrombosis in ET. Indeed, if these results are validated on other datasets, routine testing for blood group type in ET patients may represent a cheap and easily available test that could help clinicians worldwide to identify patients at high risk of venous thrombosis. We hope that, in the near future, these analyses will also extend to patients with other chronic myeloproliferative neoplasms (MPNs).

As we already mentioned, a significant proportion of ET patients with B‐type blood group in the study by Karrar et al. [1] were treated with cytoreduction (83%), antiplatelet (73%), and anticoagulant treatment (48%), calling for other possible explanations for the increased thrombotic risk in this particular subset of patients. Considering that several prior studies have already demonstrated an increased risk of venous thromboembolism in persons with non‐O blood types [8, 9, 10], we would like to suggest to the authors to analyze whether DARC‐negative phenotype may have been associated with higher thrombotic risk in their study [1]. As MPNs are characterized by constitutive leukocyte activation and chronic inflammatory state which are both, at least partly, responsible for the increased thrombotic risk [11, 12], this particular erythrocyte phenotype may accentuate inflammation and increase leukocyte trafficking through vascular endothelium thus having the potential to promote thrombosis in all ET patients not only in those with B‐type blood group. We hope that the authors can provide such an analysis.

## AUTHOR CONTRIBUTIONS

Both authors conceptualized, wrote, and approved the final version of the manuscript.

## CONFLICT OF INTEREST STATEMENT

Both authors declare no conflict of interest.

## FUNDING INFORMATION

None.

## ETHICS APPROVAL STATEMENT

The authors have confirmed ethical approval statement is not needed for this submission

## PATIENT CONSENT STATEMENT

The authors have confirmed patient consent statement is not needed for this submission

## CLINICAL TRIAL REGISTRATION

The authors have confirmed clinical trial registration is not needed for this submission.

## Data Availability

No data was generated for this article.
